# MultiChIPmixHMM: an R package for ChIP-chip data analysis modeling spatial dependencies and multiple replicates

**DOI:** 10.1186/1471-2105-14-271

**Published:** 2013-09-09

**Authors:** Caroline Bérard, Michael Seifert, Tristan Mary-Huard, Marie-Laure Martin-Magniette

**Affiliations:** 1 INRA, UMR 518 MIA, F-75005 Paris, France; 2 AgroParisTech, UMR 518 MIA, F-75005 Paris, France; 3 INRA, UMR 1165 URGV, Evry, France; 4 UEVE, UMR URGV, Evry, France; 5 CNRS, ERL 8196 UMR URGV, Evry, France; 6 Université de Rouen, LITIS EA 4108, Mont-Saint-Aignan, France; 7 UMR de Génétique Végétale, INRA, Université Paris-Sud, CNRS, Gif-sur-Yvette, France; 8 Innovative Methods of Computing, Center for Information Services and High Performance Computing, Technical University Dresden, Dresden, Germany; 9 Cellular Networks and Systems Biology, Biotechnology Center, Technical University Dresden, Dresden, Germany

## Abstract

**Background:**

Chromatin immunoprecipitation coupled with hybridization to a tiling array (ChIP-chip) is a cost-effective and routinely used method to identify protein-DNA interactions or chromatin/histone modifications. The robust identification of ChIP-enriched regions is frequently complicated by noisy measurements. This identification can be improved by accounting for dependencies between adjacent probes on chromosomes and by modeling of biological replicates.

**Results:**

MultiChIPmixHMM is a user-friendly R package to analyse ChIP-chip data modeling spatial dependencies between directly adjacent probes on a chromosome and enabling a simultaneous analysis of replicates. It is based on a linear regression mixture model, designed to perform a joint modeling of immunoprecipitated and input measurements.

**Conclusion:**

We show the utility of MultiChIPmixHMM by analyzing histone modifications of *Arabidopsis thaliana*. MultiChIPmixHMM is implemented in R and including functions in C, freely available from the CRAN web site: http://cran.r-project.org.

## Background

Chromatin immunoprecipitation coupled with hybridization to a tiling array (ChIP-chip) is a cost-effective and routinely used method for identifying target genes of transcription factors, for analyzing histone modifications or for studying the methylome on a genome-wide scale [1]. In a ChIP-chip experiment, a chromatin immunoprecipitation sample (IP) is compared against a reference sample of genomic DNA (Input). In recent years, different methods for the identification of ChIP-enriched regions have been developed. Among them, [2] proposed a linear regression mixture model named ChIPmix, designed to perform a joint modeling of IP and Input measurements. This two-component mixture model discriminates the population of enriched probes from non-enriched ones. Over the last years, ChIPmix has successfully been applied to the identification of methylated gene promoters, histone modifications or transcription factor target genes (e.g. [3-7]). However, ChIPmix has basically two important limitations: it does not model spatial dependencies between adjacent probes on chromosomes and it also does not handle the joint analysis of multiple biological replicates.

Here, we present MultiChIPmixHMM for ChIP-chip analyses enabling modeling of spatial dependencies and a simultaneous analysis of replicates to further improve the identification of enriched probes. We demonstrate improved performance of MultiChIPmixHMM compared to ChIPmix for the target identification of the chromatin mark H3K27me3 of the model plant *Arabidopsis thaliana*.

## Implementation

MultiChIPmixHMM is based on a two-state first-order Hidden Markov Model (HMM) with state-specific Gaussian emission distributions modeling immunoprecipated signals as a linear regression of reference input signals. Let (*x*_*tr*_,*y*_*tr*_) be the pair of log-Input and log-IP intensities of probe *t* measured in replicate *r* of a ChIP-chip experiment. The hidden state of probe *t* is modeled by *z*_*t*_∈{0,1} to distinguish enriched (*z*_*t*_=1) from non-enriched probes (*z*_*t*_=0). The Gaussian emission density of state *z*_*t*_ modeling *R* replicates is given by a product of independent Gaussian distributions

$$\begin{array}{l} f(y_{t1},\ldots ,y_{\text{tR}}|z_{t})=\overset{R}{\underset{r=1}{\prod }}N(a_{z_{t}r}+b_{z_{t}r}x_{\text{tr}},\;\sigma_{r}^{2}) \end{array}$$

with specific mean $a_{z_{t}r}+b_{z_{t}r}x_{\text{tr}}$ and variance $\sigma_{r}^{2}$ for each replicate *r*∈{1,…,*R*}. Dependencies between adjacent genomic probes *t* and *t*+1 are modeled by a first-order Markov chain defining that the next state *z*_*t*+1_ is depending on the predecessor state *z*_*t*_. All parameters of the HMM are estimated using the Baum-Welch algorithm [8] representing a special case of the EM algorithm [9]. To obtain relevant initial values of the emission distribution parameters (slopes and intercepts of the regressions), we applied a Principal Component Analysis to each biological replicate and used the first axis to derive the intercept and slope of the regression. All initial transition parameters are set to 0.5. This reflects the typical case where no biological information is available. We observed on simulations that alternative choices for the transition matrix initialization lead to similar results (not shown). Identification of enriched probes is based on conditional probabilities. A probe is declared enriched if its enriched conditional probability (state-posterior probability of the enriched state) is higher than 1−*α*, where *α* is chosen by the user. This strategy has been proved to yield in controlling the proportion of misclassification in mixture models [10].

## Results and discussion

### Simulations

In this section, we first compare ChIPmix, MultiChIPmixHMM and TileHMM [11], which is a method based on an HMM model to analyze the logratios (IP over Input). Moreover TileHMM can handle multiple replicates. We simulated data according to a two-state HMM with state-specific Gaussian emission distributions modeling immunoprecipated signals as a linear regression of reference input signals. We considered two test scenarios: (i) well-separated non-enriched and enriched probes (slope parameters 0.6 and 0.99) and (ii) overlapping populations of non-enriched and enriched probes (slope parameters 0.5 and 0.65). Two biological replicates are simulated for each scenario. The transition matrix is set to $\left(\begin{array}{cc} 0.97 & 0.03\\ 0.1 & 0.9 \end{array}\right)$ and the variances are set to 0.7 for the first replicate and 0.75 for the second. We used the corresponding method-specific conditional probabilities for probes to be enriched to display ROC curves. For ChIPmix, that returns a set of probe conditional probabilities per replicate, we summarized the results by taking either the minimal (resp. maximal) conditional probabilities over the two replicates.

**Figure 1:**
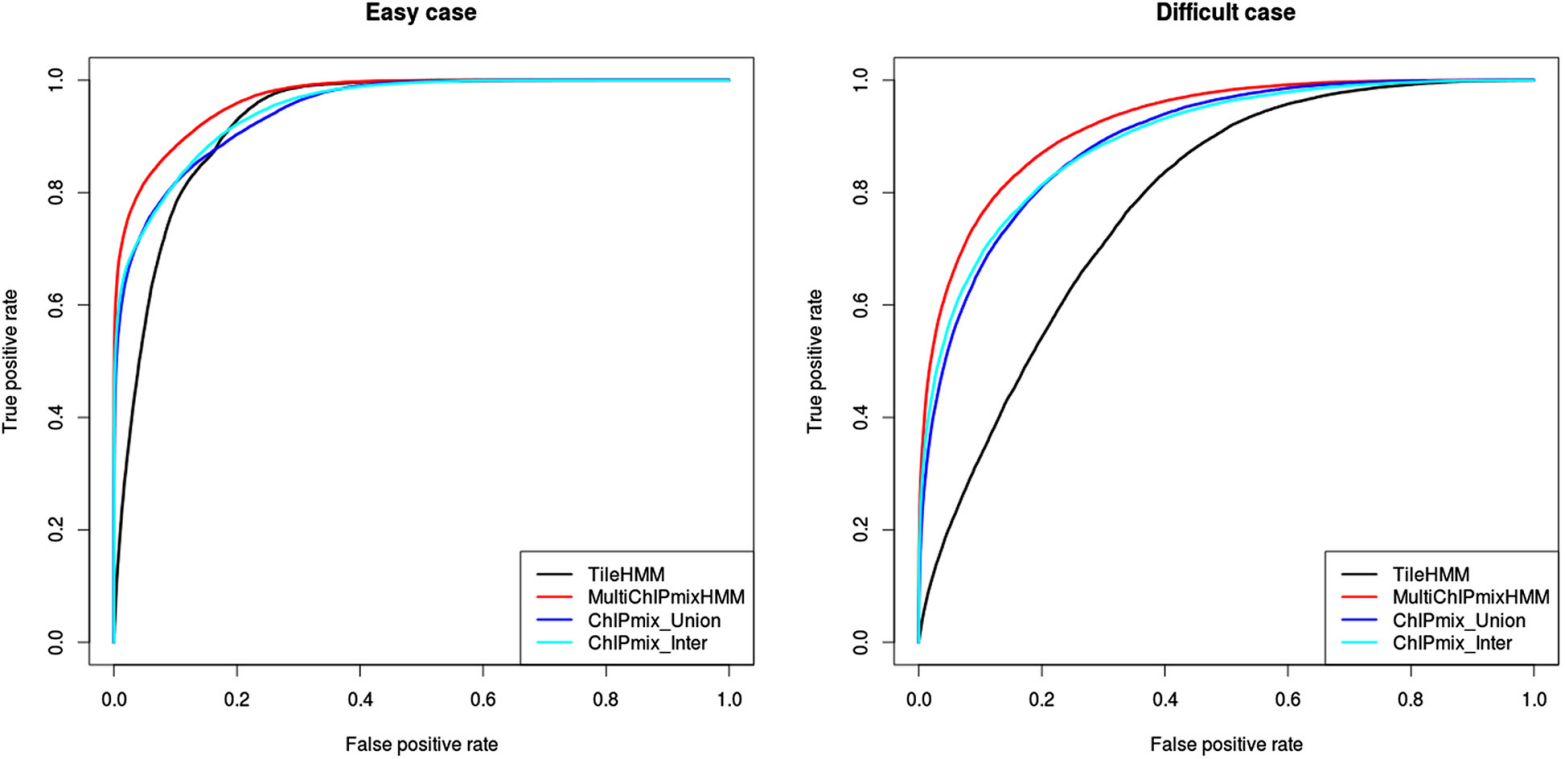
**ROC curves.** ROC curves for ChIPmix, MultiChIPmixHMM and TileHMM, for the two simulated scenarios. In ChIPmix_Union, the minimal value of the conditional probability over the replicates is considered. In ChIPmix_Inter, this is the maximal value.

On the ROC curves, we can observe that MultiChIPmixHMM outperforms the other methods whatever the scenario (cf. Figure 1). We further analyse the results after classification by choosing a level *α*=0.01.

The comparison is performed in Table 1. While conservative, ChIPmix and MultiChIPmixHMM correctly control the proportion of FP at the required 0.01 level. On the contrary, TileHMM results in a higher TP rate, but to the price of a FP rate ten time higher than the required level.

**Table 1:** Comparison of ChIPmix, MultiChIPmixHMM and TileHMM after classification

**Scenario 1, classification with *α = 0.01***		
	**false positive rate**	**true positive rate**
ChIPmix Union	3.79e-04	0.32
ChIPmix Intersection	0	0.1
MultiChIPmixHMM	1.12e-04	0.42
TileHMM	0.13	0.83

### Arabidopsis dataset analysis

To illustrate the benefit of using MultiChIPmixHMM compared to standard ChIPmix, we use a normalized ChIP-chip data set of the model plant *Arabidopsis thaliana* by [6] to compare the identification of genomic regions marked by histone H3 tri-methylated at lysine 27 (H3K27me3). We applied both methods to analyze the two biological replicates and identified probes enriched in H3K27me3 using a stringent cutoff of 1−*α*=0.99. Since ChIPmix does not handle multiple replicates, both replicates were analyzed separately and only probes declared as enriched in both replicates were finally considered as enriched (considering probes declared enriched for at least one of the replicates leads to similar results).

**Figure 2:**
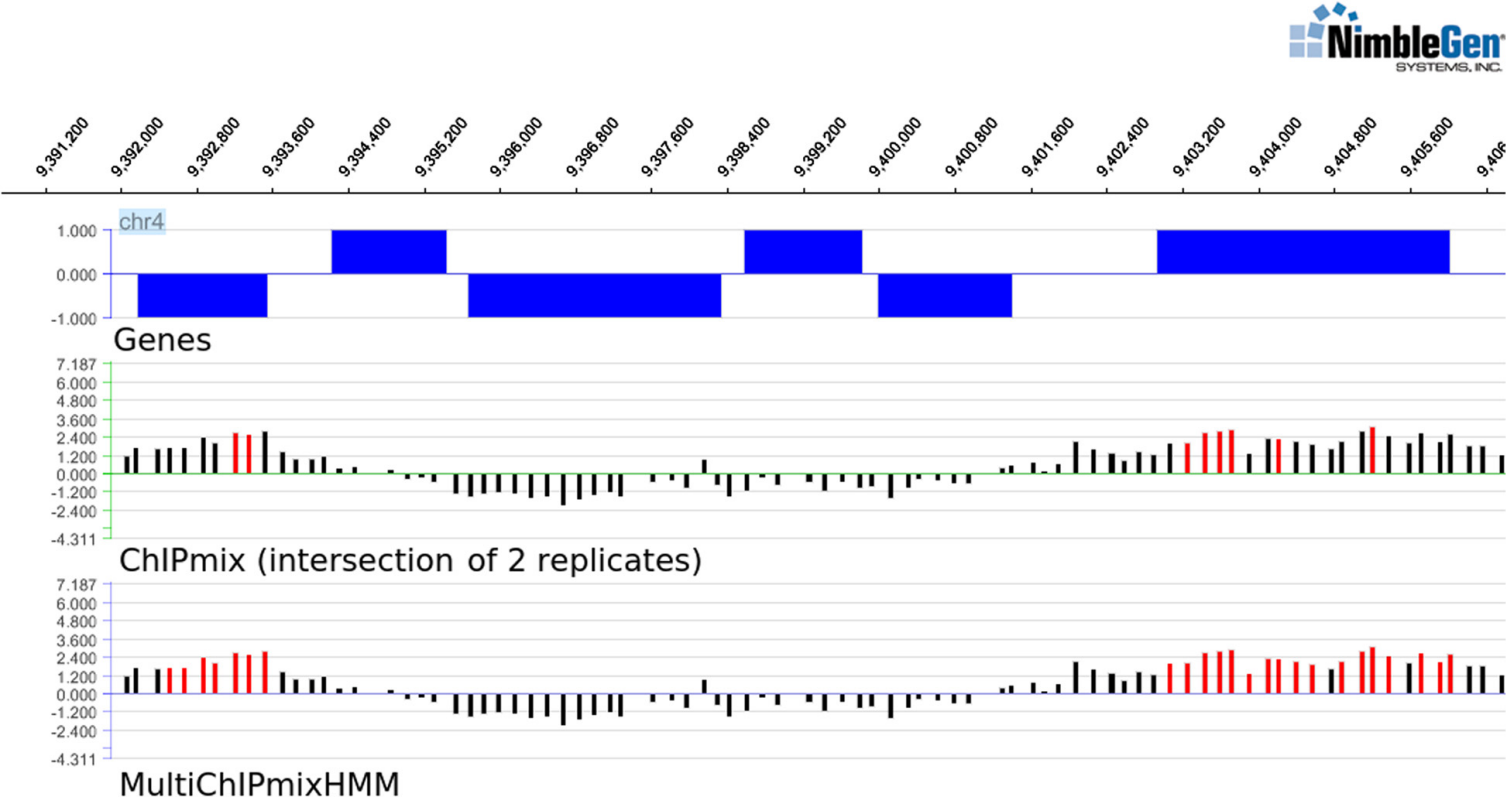
**Comparison of ChIPmix and MultiChIPmixHMM.** Comparison of ChIPmix and MultiChIPmixHMM illustrated for a selected region on chromosome 4. Probes identified as enriched are shown in red. Non-enriched probes are displayed in black. Blue bars correspond to the location of genes.

**Figure 3:**
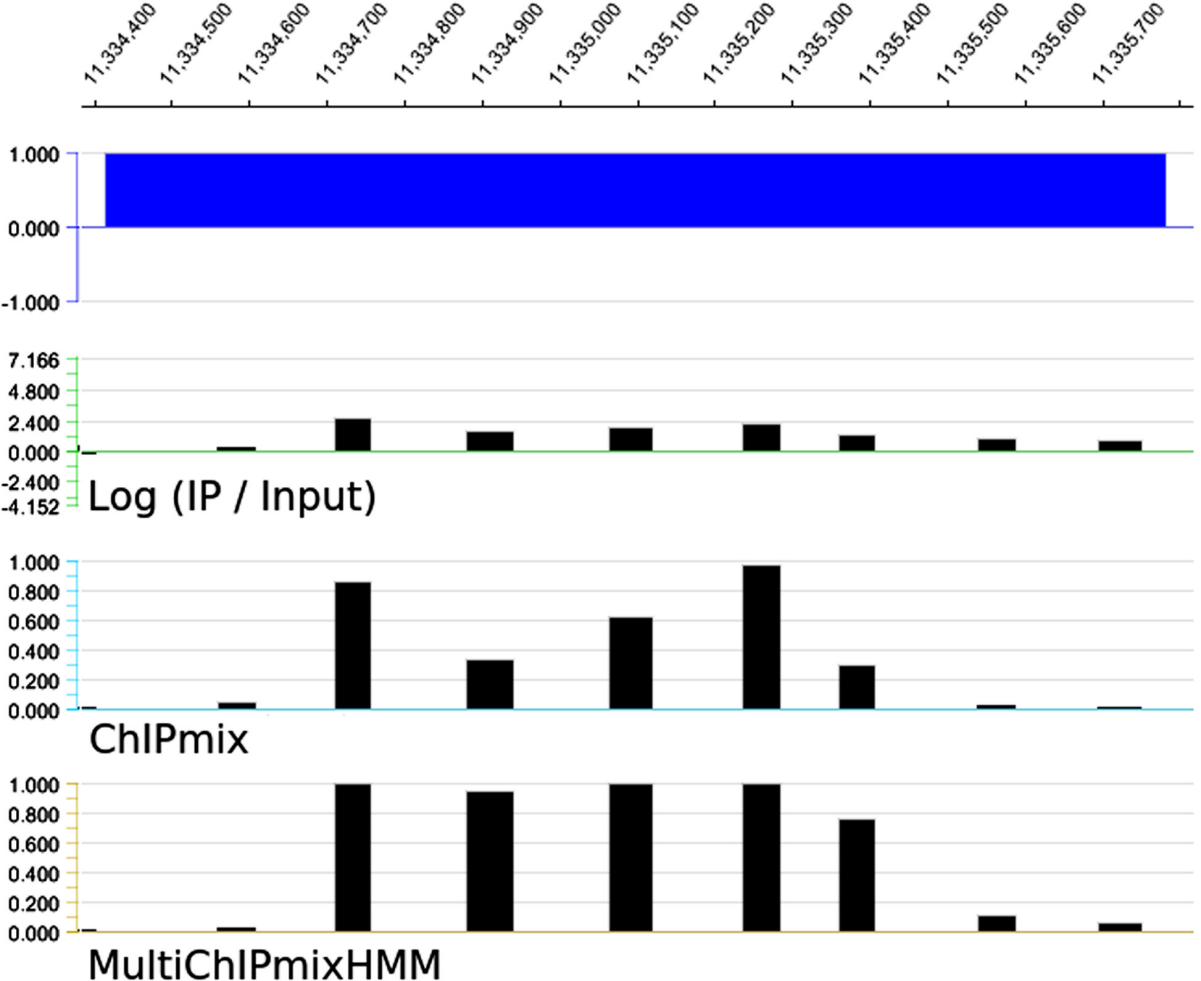
**Comparison of ChIPmix and MultiChIPmixHMM.** Example of one known H3K27me3 target gene identified only with MultiChIPmixHMM. The second line corresponds to the log-ratio signal, and for the two last lines, the scale corresponds to the conditional probabilities. We note that the conditional probabilities are clearly higher with MultiChIPmixHMM.

Considering the decodings of individual probes, ChIPmix and MultiChIPmixHMM provide the same status prediction (non-enriched or enriched) for more than 90% of the probes. Focusing on enriched probes, all the 8,100 probes identified by ChIPmix are also included in the set of enriched probes identified by MultiChIPmixHMM. MultiChIPmixHMM also identified 7,940 additional probes enriched in H3K27me3. In good agreement with previous findings [6], we find that probes marked by H3K27me3 are preferentially associated with genes. ChIPmix found about 3000 enriched probes associated with genes while there are approximately 2000 more for MultiChIPmixHMM. Among these 2000 additional probes, about 1500 complete regions already found by ChIPmix, while 536 probes concern 254 new genes. We further analyzed the identified 379 genes targeted by H3K27me3 that have been identified by both methods. Considering MultiChIPmixHMM, these genes are covered by 1616 enriched probes compared to only 939 enriched probes identified by ChIPmix. Thus, the modeling of spatial dependencies between probes by MultiChIPmixHMM leads to a better modeling of enriched probes along genes. Furthermore, MultiChIPmixHMM identified 254 new target genes. This is exemplarily illustrated in Figures 2 and 3, where additional probes identified as enriched by MultiChIPmixHMM extend or complete enriched regions identified by ChIPmix.

To further validate these findings, we use known H3K27me3 target genes based on independent prior studies by [12] and by [13]. Among the 311 genes found by both studies, 298 were commonly identified by ChIPmix and MultiChIPmixHMM. Additionally, MultiChIPmixHMM identifies 11 genes exclusively, which have already been identified as target genes in at least one of the two studies. Importantly, this increase of detection power comes without an additional computational time, because the main algorithm of MultiChIPmixHMM is implemented in *C*.

## Conclusions

The R package MultiChIPmixHMM implements a linear regression mixture model to analyse ChIP-chip data. In order to provide a more accurate identification of enriched probes, it enables to take into account spatial dependencies between directly adjacent probes and a simultaneous analysis of replicates. The benefits of MultiChIPmixHMM have been shown by analyzing both simulated and real datasets, and by comparing competing softwares.

## Availability and requirements

MultiChIPmixHMM is publicly available as an R package from CRAN [14]. Two functions are implemented and refer to the models describe before. To distinguish between the model and the function, the first letter of the name of the function is a lower case: (i) multiChIPmixHMM for modeling spatial dependencies and multiple replicates and (ii) multiChIPmix to model multiple replicates ignoring spatial dependencies between probes. Both functions take as input a vector of filenames (one biological replicate per file), and display as output a file containing the enriched conditional probability and status of each probe. 

• **Project name:** MultiChIPmixHMM

• **Project home page: **http://cran.r-project.org/web/packages/MultiChIPmixHMM/index.html

• **Operating system(s):** platform independent

• **Programming language:** R and C

• **Other requirements:** No

• **License:** GNU GENERAL PUBLIC LICENSE

• **Any restrictions to use by non-academics:** it is available for free download.

## Competing interests

The authors declare that they have no competing interests.

## Authors’ contributions

CB made the R package, analyzed the case study and drafted the manuscript. MS identified and helped to prepare the case study and drafted the manuscript. M-LM-M and TM-H designed the study and proposed the modelings. All authors helped to data analysis, to draft the manuscript, and approved the final manuscript.

## References

[B1] Buck M Lieb J ChIP-chip : considerations for the design, analysis, and application of genome-wide chromatin immunoprecipitation experiments Genomics 2004 83 3 349 360 10.1016/j.ygeno.2003.11.004 14986705

[B2] Martin-Magniette M Mary-Huard T Bérard C Robin S ChIPmix: mixture model of regressions for two-color ChIP-chip analysis Bioinformatics 2008 24 i181 i186 10.1093/bioinformatics/btn280 18689822

[B3] Kubo A Suzuki N Genomic cis-regulatory networks in the early Ciona intestinalis embryo Development 2010 137 1613 1623 10.1242/dev.046789 20392745

[B4] Long T Tsukagoshi H The bHLH transcription factor POPEYE regulates response to Iron deficiency in Arabidopsis roots Plant Cell 2010 22 2219 2236 10.1105/tpc.110.074096 20675571 PMC2929094

[B5] Moghaddam A Roudier F Seifert M Bérard C Additive inheritance of histone modifications in Arabidopsis thaliana intra-specific hybrids Plant J 2011 67 4 691 700 10.1111/j.1365-313X.2011.04628.x 21554454

[B6] Roudier F Ahmed I Bérard C Sarazin A Mary-Huard T Integrative epigenomic mapping defines four main chromatin states in Arabidopsis EMBO J 2011 30 1928 1938 10.1038/emboj.2011.103 21487388 PMC3098477

[B7] Seifert M MeDIP-HMM: Genome-wide identification of distinct DNA methylation states from high-density tiling arrays Bioinformatics 2012 28 22 2930 2939 10.1093/bioinformatics/bts562 22989518

[B8] Rabiner L A tutorial on hidden markov models and selected applications in speech recognition Proc IEEE 1989 77 257 286 10.1109/5.18626

[B9] Dempster AP Laird NM Rubin DB Maximum likelihood from incomplete data via the EM algorithm J R Stat Soc, series B 1977 39 1 38

[B10] Mary-Huard T Error rate control for classification rules in multi-class mixture models Journées de la société française de statistique. SFDS Proceedings; 2013 Toulouse

[B11] P Humburg DB Stone G Parameter estimation for robust HMM analysis of ChIP-chip data BMC Bioinformatics 2008 9 343 10.1186/1471-2105-9-343 18706106 PMC2536674

[B12] Turck F Arabidopsis tfl2/lhp1 specifically associates with genes marked by trimethylation of histone h3 lysine 27 PLoS Genet.v 2007 3 6 e86 10.1371/journal.pgen.0030086 PMC1885283 17542647

[B13] Zhang X Whole-genome analysis of Histone H3 Lysine 27 Trimethylation in Arabidopsis PLoS Biol 2007 5 5 e129 10.1371/journal.pbio.0050129 17439305 PMC1852588

[B14] Team RDC A language and environment for statistical computing 2013 Vienna, Austria: R Foundation for Statistical Computing ISBN(3-900051-07-0)

